# Peanuts, Aflatoxins and Undernutrition in Children in Sub-Saharan Africa

**DOI:** 10.3390/nu9121287

**Published:** 2017-11-26

**Authors:** Innocent Mupunga, Pamella Mngqawa, David R. Katerere

**Affiliations:** 1 Department of Biomedical Sciences, Tshwane University of Technology, Pretoria 0183, South Africa; mupunga@gmail.com; 2 Mycotoxicology and Chemoprevention Research Group, Institute of Biomedical and Microbial Biotechnology, Cape Peninsula University of Technology, Cape Town 7535, South Africa; MngqawaP@cput.ac.za; 3 Department of Pharmaceutical Sciences, Tshwane University of Technology, Pretoria 0183, South Africa

**Keywords:** aflatoxins, malnutrition, peanuts, food safety, weaning foods

## Abstract

Peanuts (*Arachis hypogaea*) is an important and affordable source of protein in most of Sub-Saharan Africa (SSA) and a popular commodity and raw material for peanut butter, paste and cooking oil. It is a popular ingredient for foods used at the point of weaning infants from mother’s milk. It is at this critical point that childhood undernutrition occurs and the condition manifests as stunting, wasting and growth restriction and accounts for nearly half of all deaths in children under five years of age in SSA. Undernutrition is multi-factorial but weaning foods contaminated with microbiological agents (bacteria and fungi) and natural toxins have been shown to play a big part. While peanuts may provide good nutrition, they are also highly prone to contamination with mycotoxigenic fungi. The high nutritive value of peanuts makes them a perfect substrate for fungal growth and potential aflatoxin contamination. Aflatoxins are highly carcinogenic and mutagenic mycotoxins. This article reviews the nutritional value and aflatoxin contamination of peanuts, the role they play in the development of childhood malnutrition (including the different theories of aetiology) and immunological problems in children. We also discuss the control strategies that have been explored and advocacy work currently taking shape in Africa to create more awareness of aflatoxins and thus combat their occurrence with the goal of reducing exposure and enhancing trade and food safety.

## 1. Introduction

In sub-Saharan Africa (SSA), one in seven children die before their fifth birthday [1]. Nearly half of these under five deaths are attributable to underlying undernutrition which manifests as stunting, wasting and growth restriction in utero and micronutrient deficiencies [2]. Wasting is an indicator of acute malnutrition and is defined as inadequate weight for age and height while stunting which is an indicator of the cumulative effects of chronic undernutrition and infection is defined as inadequate height for age [2,3]. According to the United Nations, nearly 25% (or 162 million) of children under the age of five globally had stunted growth in 2014 [3]. SSA and Southern Asia accounts for more than 75% of these stunted children and more than 90% of all the under five deaths [1,3]. Apart from death, the consequences of undernutrition are wide and include substantial morbidity, loss of quality of life, long-term developmental problems, and poor performance in school which leads to diminished ability to work, hence reducing the potential for contribution towards national development [1,4]. Thus, the economies of SSA are partly affected by a labour force that is hobbled by the effects of poor nutrition in childhood.

Development of childhood malnutrition usually coincides with the introduction of complementary foods during weaning [5]. Most of the complementary foods are usually nutritionally deficient with poor nutrient density and diversity, and fail to meet the dietary demands for the development of an infant into a child [6]. These complementary foods may also be contaminated by microbiological agents (bacteria and fungi), and environmental toxins (bacterial toxins; fungal toxins such as mycotoxins; metals and their complexes; and organic chemicals) [6]. Infants are also exposed to contaminated water, poor sanitation and hygiene which result in ingestion of microbes leading to high rates of diarrhoea and other infections that damage the small intestine. Damaged intestines are characterized by altered barrier integrity, mucosal inflammation and reduced nutrient absorption, which all contribute to malnutrition [6].

There seems to be a relationship between malnutrition, poverty and food security and safety [6]. Food security and safety interventions can influence nutrition and growth in young children by providing adequate micronutrients and dietary diversity, as well as eliminating microbial contaminants. This will lead to reduced exposure to environmental chemicals and toxins and reduced cases of diarrhoea, all from foodborne pathogens. Promotion of nutrient rich complementary weaning foods (e.g., peanuts) while discouraging over-reliance on carbohydrate dense staples is also recommended to prevent malnutrition in weaned children [2]. Research has shown dietary diversity to be associated with micronutrient adequacy and better anthropometric status of children [5].

## 2. Nutritional Value of Peanuts

Peanuts, which are sometimes called poor men’s food, are affordable and adaptable to a variety of culinary uses making them the most consumed nut in most SSA regions [7]. They can be consumed raw, boiled, roasted or mixed with other dishes such as vegetables, porridge, and meat, and spread on bread [8]. In nutritional terms, peanuts are ranked sixth among oil producing crops and eighth among nutritional crops [9]. They are rich in nutrients (carbohydrates, lipids, proteins, vitamins, minerals, fibre and some organic acids) that are essential to human health and are adequate to meet energy and protein nutritional needs of populations at risk of malnutrition (see Table 1) [10,11]. They contain all the essential amino acids (lysine, leucine, isoleucine, tryptophan, threonine, phenylalanine, methionine, tyrosine, valine and histidine) making them a critical component of the human diet especially in communities where animal derived protein sources are not readily available [10,11]. The peanut protein is plant based, most of the fat is unsaturated and the fibre is a complex carbohydrate which makes peanuts the best form of human nutrition [11]. Since peanuts are legumes, they have higher protein content compared to other nuts, with levels comparable to beans [11].

A number of compounds (e.g., coenzyme Q10, several B group vitamins, vitamin E and antioxidant minerals) with added health benefits beyond basic nutrition have been identified in peanuts and their skins [11]. Consumption of peanuts or peanut oil has been linked to reduced risk of cardiovascular disease. That is achieved through probable improvement in serum lipid profile with decreased low-density lipoprotein (LDL) cholesterol oxidation that exerts a cardiovascular protective effect [11,12]. Peanuts also contribute to reduced risk of blood pressure, diabetes, Alzheimer’s, gallstones and obesity [11].

In most Southern African countries, peanuts and their products are used as weaning food for children, supplementing diets where maize is the major energy staple food [9,13]. In a study in Eastern Zimbabwe, about 54% of the farmers interviewed cultivated peanuts for making peanut butter while another 14% cultivated peanuts for extracting oil [14]. The peanut butter is used in porridge, sandwiches and as a sauce in vegetables and other relishes while the extracted oil is used for cooking, replacing the more expensive commercial cooking oil. Thus, peanuts are an important nutritional source in many rural communities especially among infants and children who are most vulnerable to malnutrition. Peanuts are ground up into butter which is then mixed with maize meal porridge and commonly used for infant feeding. Peanut butter is also used as the main ingredient in ready-to-use therapeutic food (RUTF), an energy dense paste that does not require cooking and can be stored for months [15]. In Malawi, RUTF was found to be a more effective supplementary feed compared to the fortified corn/soy supplement [15].

In recent years, peanut allergy has become a major concern. There is general uncertainty about the prevalence of food allergies in general [16]. A meta-analysis of various studies has shown the prevalence to range 0–2% [16,17]. No peanut consumption studies in Africa were readily available in the literature and studies on prevalence of peanut allergies are few and far between [18].

However, studies have shown that early exposure to peanuts in infancy is associated with lower allergy prevalence, so the popularity of peanut-based weaning foods in Africa may work to reduce peanut allergies [19].

## 3. Aflatoxin Contamination of Peanuts

The high nutritive value of peanuts makes them a perfect substrate for fungal growth and potential mycotoxin contamination. Aflatoxins are a highly carcinogenic and mutagenic group of mycotoxins produced by *Aspergillus* species, mainly *A. flavus* and *A. parasiticus* [20]. *A. flavus* and *A. parasiticus* are abundant in the tropical and subtropical regions, the same regions where peanuts are primarily produced [21]. Hot and humid climates, prevalent in the tropics promote fungi which infect the crop in the field and/or immediately after harvest during storage [22]. Drought stress, and insect damage increase the susceptibility of the crops to fungal infection leading to contamination before harvest while improper drying and poor storage conditions lead to contamination in storage facilities [20]. In some African communities, peanuts are traditionally shelled by hand, a painful and time consuming task but, to ease the process, the peanuts are soaked in water to soften the shells then shelled and stored without proper drying before being taken to the market [23]. The moisture introduced during shelling and improper drying creates a conducive environment for fungal proliferation and aflatoxin production.

There are four major aflatoxins, aflatoxin B1 (AFB1), AFB2, AFG1, and AFG2, of which AFB1 is the most potent natural carcinogen and mutagen known and has also been classified as a Group 1 human carcinogen by the International Agency for Research on Cancer [22,24]. Several studies conducted in the last 10 years point to a widespread problem of aflatoxin contamination in peanuts in many Southern African countries including Zimbabwe [25], The Democratic Republic of Congo (DRC) [7,8], South Africa [7], Zambia [26,27], and Malawi [9]. Weaned children are more vulnerable to aflatoxicosis since their diet is monotonous, alternating between breast milk and weaning foods like maize porridge and local vegetables, usually served with a dash of peanut butter [28]. The level of contamination found in these countries as reported in the studies generally exceeded the maximum regulatory limits for peanuts and peanut products meant for human consumption set by the Codex Alimentarius at 5 µg/kg (AFB1) and 20 µg/kg (total) which SSA countries also adhere to in their national protocols [25].

Njoroge et al. conducted a comprehensive multi-year analysis of aflatoxin contamination in 24 peanut butter brands sold in Zambia (locally produced and imported) between 2012 and 2014 [26]. The number of brands contaminated varied from year to year with 73% (8/11), 87% (13/15) and 53% (10/19) having AFB1 levels >20 µg/kg in 2012, 2013 and 2014, respectively. In 2013, some of the peanut butter samples were found with levels of up to 10,000 µg/kg AFB1. The brands tested included locally manufactured peanut butter and imported peanut butter from Malawi, South Africa and Zimbabwe. This implies that the problem of AFB1 contamination in peanut butter is regional even though the imported brands had significantly lower AFB1 contamination levels compared to local brands. Imported brands are usually from formal manufacturers who have quality management systems in place. The same research group also reported high levels of AFB1 in peanut kernels (up to 11,100 µg/kg) and milled peanut powder samples (up to 3000 µg/kg) collected between 2012 and 2014 from the major peanut growing regions of Zambia [27]. In Zambia, milled peanut powder is used as a paste in vegetables, mixed with cereals for making porridge and as an ingredient in complementary foods for HIV and AIDS patients.

Mupunga et al. carried out a survey to determine the extent of fungal and aflatoxin contamination of peanuts and peanut butter bought from both the formal and informal markets of Bulawayo, Zimbabwe’s second largest city [25]. Eighteen peanut and 11 peanut butter samples were purchased in total. *Aspergillus* species were isolated in 27% of the peanut butter samples with up to 100 CFU/g and 67% of the peanut samples ranging from 3–20% of the kernels examined. Of the 18 peanut samples, three (27%) were contaminated with total aflatoxins (range: 6.6–622.1 µg/kg) and AFB1 (range: 6.3–528 µg/kg). Ninety-one per cent of the peanut butter samples were contaminated with total aflatoxins, ranging 6.1–247 µg/kg, with average AFB1 levels of 55.73 µg/kg. A worrying finding from this study was that contamination levels in homemade peanut butter were similar to levels in commercial peanut butter implying that quality management systems are not being implemented or are being compromised by commercial manufacturers. It is critical to note this was a small sample size; however, these findings still show that peanuts and peanut butter from southern Zimbabwe were contaminated with unacceptably high aflatoxin levels, placing consumers at risk.

Kamika et al. compared fungal and aflatoxin occurrence in peanuts sold at informal markets from Kinshasa, Democratic Republic of Congo (DRC) and Pretoria, South Africa [7]. All samples from Pretoria and 95% (19/20) from Kinshasa were contaminated with *Aspergillus* species with total colony counts ranging 40–21,000 CFU/g and 20–49,000 CFU/g for Pretoria and Kinshasa, respectively. The level of fungal contamination was significantly higher for Kinshasa than for Pretoria (*p* < 0.001) and this was attributed to poor storage conditions and longer storage in climatic conditions conducive to fungal growth. All samples from Kinshasa and 90% (18/20) from Pretoria were contaminated with total aflatoxins ranging from 2.19–1258 µg/kg and 2.1–73.5 µg/kg for Kinshasa and Pretoria respectively. AFB1 was the predominant isomer accounting for nearly half of the total aflatoxin concentration in each sample. Overall, the samples from Pretoria had significantly lower (*p* < 0.001) AFB1 levels compared to Kinshasa samples. Kamika and Takoy also assessed AFB1 contamination levels in raw peanuts collected from the rural areas of Kinshasa, DRC during both the dry and the rainy seasons [8]. Predictably, AFB1 was detected in higher concentration and more samples during the rainy season compared to the dry season. The main limitation of this study is that the authors only report single year data, when it is known that contamination can vary between seasons depending on the rainfall received and drought stress experienced by the crops in the field [20].

In a study in Malawi, AFB1 contamination has been reported in all the 11 major peanut producing districts, with contamination levels ranging up to 2197 µg/kg and 3240 µg/kg in 2008 and 2009 respectively [9]. Forty-six per cent (46%) of the 212 peanut samples (including peanut butter) collected after 8–11 months storage in 2008 and 23% of the 1185 fresh peanut samples collected after 1–2 months in storage in 2009 were contaminated. Of these, 21% of the 2008 and 8% of the 2009 samples had AFB1 levels above 20 µg/kg. *Aspergillus* species contamination was also observed in soil samples collected from the different farms. Higher *Aspergillus* colony forming units (>3000 CFU/g) was directly correlated with an increased chance of peanut contamination with AFB1.

## 4. Aflatoxins and Malnutrition

Several nutritional and growth effects have been observed in animal studies where several animal species including ducks, ducklings, mice, quail, pigs and fish were exposed to aflatoxins. These include reduced feed intake, poor weight gain, and decreased food conversion rate in older animals [29]. Growth retardation including reduced body weight, delayed physical and behavioural development pre-weaning and poor locomotor coordination and other impairments post-weaning have been observed in baby animals whose mothers were exposed to aflatoxins during pregnancy [29]. These deleterious effects in animals are mirrored in humans exposed to aflatoxins.

Children in sub-Saharan Africa are exposed to aflatoxins very early in life including in utero through maternal food intake, during breastfeeding, through weaning and post-weaning periods through foods like peanuts and maize [29,30]. Most of these children are exposed to high aflatoxin levels throughout their lives since most communities rely on subsistence farming and have little appreciation of the presence of mycotoxins (or alternative food options for that matter). Most of the subsistence farmers have limited dietary diversity and little or no interventions to control aflatoxin contamination [31]. Evidence from West Africa at the beginning of the millennia showed that chronic exposure to aflatoxins in children under five was linked to poor growth (underweight and stunted) and poor immune status [32,33]. In this region, weaning status was associated with high levels of aflatoxin exposure, suggesting contamination of weaning foods with aflatoxins, which led to growth faltering, particularly stunting [32].

Turner et al. investigated the relationship between aflatoxin exposure in utero and growth faltering in Gambian children [30]. All the maternal blood samples collected during pregnancy had detectable levels of aflatoxin–albumin adducts, implying all mothers enrolled in the study were exposed to aflatoxins during pregnancy. Nearly half of the cord blood samples had detectable aflatoxin-albumin adduct levels, and these were significantly correlated (*p* < 0.001) with maternal aflatoxin-albumin levels. Higher maternal aflatoxin-albumin levels were significantly correlated (−0.249 SD, *p* = 0.012) to lower weight for age, and also significantly correlated (−0.207 SD, *p* = 0.044) to stunting. The presence of aflatoxin metabolites in cord blood as well as the growth faltering observed provide evidence that aflatoxins can cross the maternal placental barrier exposing the unborn child to biochemical toxicities whose effects become apparent in early infancy. Similarly, in Ghana, the risk for low birth weight increased with increasing levels of aflatoxin exposure in pregnant women, however this was a cross-sectional study with limited ability to draw causal or temporal relationships [34].

Chronic aflatoxin exposure has also been linked with kwashiorkor, a severe Protein Energy Malnutrition (PEM) disease. Studies carried out in the last three decades have shown higher aflatoxin levels in the blood and urine of children with kwashiorkor compared to healthy children or children with other forms of PEM (e.g., marasmus) [29]. AFB1 was detected in the urine and blood of malnourished children with kwashiorkor and marasmic kwashiorkor in a four-year study conducted in Cameroon [35]. A link was established (*p* < 0.05) between kwashiorkor and the presence of AFB1 in urine when comparing malnourished children and the control group. In a study in Nigeria, aflatoxins were detected more frequently in patients and at higher concentrations in children with kwashiorkor and marasmic kwashiorkor compared to control groups [36]. An Egyptian study found that aflatoxins and their metabolites were more prevalent with a significantly higher serum concentration in Kwashiorkor patients than in Marasmic patients while no aflatoxins were detected in control patients [37]. Aflatoxins are known to inhibit protein synthesis and immune factors and exposure to aflatoxins may delay recovery from kwashiorkor even if the aflatoxins did not cause the condition [38].

Leeroy et al. analysed the socio-economic determinants of aflatoxin exposure. All the predictors of poverty were associated with high levels of aflatoxin exposure [39]. These include lack of disposable income for household expenditure, food insecurity and severe hunger, lack of capacity to procure and use inorganic fertilizer and pesticides, owning very little land or none at all and a lack of education. Households that had no disposable income were facing food safety and insecurity issues, were most likely to consume visibly mouldy food whether self-produced or bought from the market [14,39]. There is a high probability that farmers who can afford inorganic fertilizer and pesticides also practice good agricultural practices which limit fungal and aflatoxin contamination in the fields, post-harvest and in storage. On the contrary, poorer farmers might not have the knowledge or the capacity to implement good agricultural practices, hence their crops might be contaminated at every stage of the value chain, i.e., in the fields due to drought stress and pest infection, post-harvest due to improper drying, or poor storage practices that allow pest infestation and moisture build-up which encourage fungal growth and aflatoxin production [20,39].

Children from poor households are usually fed diets that are deficient in essential micro- and macronutrients and also lack clean drinking water and proper sanitation facilities. This leaves them more prone to diarrhoeal diseases, pneumonia, malaria, measles and other infections [29,39]. Leeroy et al. concluded that the growth stunting observed in children exposed to aflatoxins might be partially due to the confounding effects of their socioeconomic status especially poverty [39]. In the same vein, the association with kwashiorkor may be more complex: a systems analysis approach to malnutrition is probably more appropriate due to the multi-factorial nature of vulnerability. It has also been observed elsewhere that the proportion of stunted children is directly correlated with the proportion of the population living below the poverty line and inversely correlated to the gross domestic product [29]. Even though the role aflatoxins play in the pathogenesis of kwashiorkor remains unclear, the common symptoms, and the fact that aflatoxicosis and kwashiorkor occurrence follow similar geographic, socio-economic and climatic factors, point to a possible causal role [5].

## 5. Aflatoxins and Immunity

It is not clear how aflatoxin exposure results in malnutrition (stunting, growth faltering and kwashiorkor). Several mechanisms have been proposed:

(i) Aflatoxins have been shown to damage the intestinal tract leading to impaired intestinal barrier function and malabsorption and consequently zinc deficiency which causes growth faltering and immune dysfunction [40].
(ii) Enterocyte damage and increased intestinal permeability leading to systemic immune activation. The changes in intestinal integrity induced by aflatoxins may leave the hosts with poor nutritional absorption capability and more vulnerable to intestinal pathogens. When combined with poor nutrition, poor sanitation and hygiene, children exposed to aflatoxins are faced with a multiplicity of assaults ranging from infection and poor nutritional absorption from poor food quality leading to growth impairment [40].
(iii) Inhibited synthesis of proteins, enzymes, clotting factors and impaired glucose metabolism, phospholipid synthesis and fatty acid synthesis [6].
(iv) Aflatoxins mediate immune system dysfunction increasing risk of infections (e.g., gastroenteritis) in children, which may lead to impaired growth from loss of energy caused by the infection or energy expended recovering from illness [6].
(v) Aflatoxins may cause growth retardation through the same mechanisms leading to malignant transformation and tumour growth [6].

Thus, central to the symptomatic effects of aflatoxin are its immunotoxic and immunosuppressant effects [41], which are well known, particularly in other mammals. Qian et al. found that one-week post exposure to AFB1, CD8^+^ lymphocytes and Natural Killer (NK) cells were decreased in rats [41]. Prolonged exposure led to an inflammatory response and apoptosis (via upregulation of cytokines and pro-inflammatory genes), and so both exposure times negatively impacted on cell-mediated immune responses. A similar conclusion was reached by Meissonnier et al. based on work done in pigs [42]. These effects were cited in support of the Gambian study by Turner et al., which found decreased IgA in the saliva of children exposed to high amounts of AFB1 [33].

Because of the immunotoxic and hepatotoxic effects discussed above, aflatoxins have also been implicated in infectious disease modulation [43]. The rate of progression from HIV infection to AIDS has been shown to be faster in low income countries compared to the developed world [44]. The suspected cause is aflatoxin induced immunosuppression since a large number of people exposed to aflatoxin are also HIV positive in most low income countries. An association between high HIV viral load count and high levels of aflatoxin exposure has been observed [45]. Both HIV virus and aflatoxins are known to be immunosuppressive agents targeting cellular immunity [43]. Aflatoxins are metabolized to the highly reactive AFB1-8, 9-epoxide in the liver, which binds to nucleic acids and proteins forming adducts [22]. The result is inhibition of nucleotides and protein synthesis [6,45] which impacts on immune cell synthesis. Apart from this, aflatoxins (as previously alluded to) also impair micronutrient utilization which also impairs the immune system leaving patients more vulnerable to opportunistic infections hence high viral loads. The liver is the most important organ for protein and immune cell synthesis and when it is compromised this affects not only immune surveillance, but also nutrition and xenobiotic (pharmaceutical) metabolic processes. Thus aflatoxin exposure may cause vulnerability to disease and also poor outcomes to pharmacotherapeutic interventions.

## 6. Aflatoxin Control Strategies

Goal number 2 of the United Nations Sustainable Development Goals (SDGs) aims to end hunger and all forms of malnutrition by 2030 through universal access to safe, nutritious and sufficient food throughout the year [3]. The World Health Organisation (WHO) targets a “40% reduction in the number of children under-5 who are stunted” by 2025 [4]. Given the need to achieve the SDGs and meet WHO targets, the weight of evidence pointing to the link between aflatoxin exposure and malnutrition and the knowledge that aflatoxins contaminate important/staple food commodities (e.g., peanuts and grains) that are a critical solution to malnutrition, it is imperative that measures to reduce aflatoxin contamination in food be put in place. Since the aflatoxin problem lies at the interphase of agriculture, health and trade, all relevant stakeholders need to be involved when strategies to reduce aflatoxin contamination in food commodities are being interrogated. It is critically important that the reduction strategies formulated are cost effective, safe and technically feasible to be used by communities burdened by the aflatoxin problem [29]. These strategies should also be standard and scalable while taking into account local resources and cultural practices [29].

Aflatoxin control strategies should encompass pre-harvest and post-harvest practices. Pre-harvest strategies may include use of genetically modified crop varieties that are resistant to aflatoxigenic fungal infection (though these may not be culturally acceptable, or affordable), reduction of crop stress in the field (through use of irrigation, fertilizer, insecticides and fungicides), and biocontrol through use of non-aflatoxigenic fungi to compete with aflatoxigenic fungi [21]. Post-harvest strategies involve proper drying of crops, sorting to remove visibly contaminated, broken and shrivelled kernels and controlling transportation and storage conditions to avoid insect damage and moisture accumulation [21,31].

Turner et al. assessed the use of improved post-harvest storage methods for peanuts in rural Guinea [31]. This involved using a package of post-harvest intervention measures introduced to farmers from 10 villages at peanut harvest time and comparisons were made with farmers in 10 neighbouring villages that used the usual storage practices. The intervention strategies included:

(i) hand sorting to remove visibly mouldy or damaged peanuts;
(ii) drying on mats to prevent moisture accumulation from the soil and also to make it easier to gather in case of unexpected rain;
(iii) complete sun drying which was confirmed by shaking the kernels;
(iv) storage in natural fibre bags for good air circulation;
(v) storage on raised wooden pallets to avoid humidity from earthen floors; and
(vi) use of insecticide to prevent pests and insects from accumulating in storage facilities.

Aflatoxin–albumin adduct levels were measured in a subsample from both groups at the beginning of the intervention and there was no difference between group means. However, at five months post-harvest, the biomarker levels in the intervention group were significantly lower (more than half) than the control group. About 20% of the individuals from the intervention villages had undetectable aflatoxin albumin adduct levels compared to only 2% from the control villages. This study showed that simple, cheap and culturally-acceptable technological interventions appropriately targeted can have substantial effects on the overall aflatoxin exposure.

Early harvesting has been suggested as a solution to reduce aflatoxin pre-harvest contamination [46]. However early harvesting results in poorer yield and poor seed grades which would directly impact farmers’ livelihoods. Further, peanut contamination usually worsens in storage where poor phytosanitary conditions and poor ventilation create ideal fungal colonization conditions. This points to the fact that more mitigation efforts should be concentrated on the post-harvest phase of the peanut value chain.

From an advocacy point of view the African Union Commission has set up the Partnership for Aflatoxin Control in Africa (PACA) which work with works with a steering committee representing farmers, consumers, research and technology organizations, healthcare and trade professionals and the private sector to combat aflatoxins at continent level and foster health, trade and food safety. One of the key result areas of the PACA strategy is facilitating the adaptation and wider adoption of available technologies and knowledge including biocontrol through use atoxigenic fungal strains like AFLA-SAFE and development of aflatoxin resistant crop varieties [47].

## 7. Recommendations

Raising awareness and public education on occurrence, health effects and prevention of mycotoxins in general and aflatoxins in particular should be prioritized in rural farming areas. These awareness programmes should also focus on how post-harvest storage practices can influence food safety among peanut farmers and consumers. This can be achieved through the use of multimedia platforms like radio and television, social media like Facebook and incorporation of education on good agricultural practices in to the curriculum of Agricultural institutes, primary and secondary school [14]. In Zimbabwe, Dube and Mutetwa found that communal farmers and other players in the peanut value chain displayed a lack of knowledge/awareness of aflatoxins, their presence in peanuts or their health effects [14]. Thus, even though 46% of them believed inadequate drying of peanuts resulted in mould formation, the farmers in the study had no idea that peanuts could be a source of disease including aflatoxicosis and allergic reactions. Agricultural Extension workers were more informed but did not seem to be continuously raising awareness around this issue. These findings mirror the perceptions of farmers in Nigeria and Tanzania who also showed low levels of knowledge of aflatoxins [13]. On the other hand, 65% (684/1053) of farmers in Malawi had some knowledge of aflatoxins and they associated aflatoxins with rotten and mouldy peanuts [9]. Most of this knowledge was acquired from other farmers, radio programmes and agricultural institutions, pointing to a critical role public education/awareness may play in addressing the aflatoxin problem.

As a result of lack of knowledge, about 63% of the Zimbabwean farmers said they will consume mouldy peanuts as long as they were not bitter [14]. The bitter taste may be caused by high levels of aflatoxins. Some of the farmers cited prevention of food wastage as the reason for consuming mouldy peanuts. This points to a case of food insufficiency surpassing food safety concerns in most poor communities [5]. Most people in these communities consume their own food with very little left over to trade and when trade does happen it is usually limited to local informal markets where quality is not managed. It has always been noted that it is difficult to prioritize food safety in communities where food security remains challenging [28].

Investing in research and development of innovative, safe and economically viable uses for contaminated products will also go a long way in reducing aflatoxin exposure risk in vulnerable communities [23]. One of the examples is production of oil from contaminated peanuts which is being piloted in Malawi. Aflatoxin contamination is then reduced to safe levels through a simple filtration process that removes proteins. This value addition to contaminated peanuts leads to a nutritious product for domestic consumption and export markets, with the potential to uplift whole communities out of poverty in most African countries.

## 8. Conclusions

Childhood malnutrition results in a number of developmental issues and these diminish one’s ability to be productive and contribute to personal and national economic development later in life. Aflatoxins contaminate peanuts and peanut products consumed by many communities in SSA. The hot and humid conditions prevalent in tropical Africa provide a conducive environment for the growth of *Aspergillus* species and aflatoxin production [20]. Aflatoxins contribute to malnutrition by interfering with intestinal integrity and hepatic metabolism. This leads to malabsorption, micronutrient deficiencies, impaired immune function, and vulnerability to gut infections, which all lead to impaired growth and malnutrition. It is therefore important that aflatoxin control and other food safety measures be considered as part of the solution to eliminate childhood malnutrition. 

Research should focus on new innovations (such as resistant peanut varieties) but also modifying socio-cultural practices in how peanuts are harvested, stored and handled. Both initiatives require lobbying and advocacy and creating awareness among African farmers and policymakers on the major contribution which peanuts can make to the socio- status economic (through enhanced trade of high quality product) and food and nutrition security of the region, but also the risk that poor quality crop poses.

In compiling this paper, it has also become apparent that there is a paucity of information regarding peanut consumption and peanut allergy among African consumers. Such data do not even exist for high peanut consuming countries such as Ghana, Nigeria and the Democratic Republic of Congo. Exposure studies and risk assessment studies cannot be done in light of these gaps. Therefore, there should be research thrust in these areas as a base for future research. 

## Figures and Tables

**Table 1:** Nutritional value of peanuts per 100 g (adapted from Arya et al. [11]).

Nutrient	Nutrient Value	Percentage of RDA
Energy	567 Kcal	29
Carbohydrates	16.13 g	12
Protein	25.80 g	46
Total Fat	49.24 g	165
Dietary Fibre	8.5 g	22
**Vitamins**		
Folates	240 μg	60
Niacin	12.066 mg	75
Pantothenic acid	1.767 mg	35
Pyridoxine	0.348 mg	27
Riboflavin	0.135 mg	10
Thiamin	0.640 mg	53
Vitamin E	8.33 mg	55.5
**Electrolytes**		
Sodium	18 mg	1
Potassium	705 mg	15
**Minerals**		
Calcium	92 mg	9
Copper	1.144 mg	127
Iron	4.58 mg	57
Magnesium	168 mg	42
Manganese	1.934 mg	84
Phosphorus	76 mg	54
Selenium	7.2 μg	13
Zinc	3.27 mg	30
